# The range of peripapillary retinal nerve fibre layer and optic disc parameters, in children aged up to but not including 18 years of age who were born prematurely: protocol for a systematic review

**DOI:** 10.1186/s13643-016-0319-0

**Published:** 2016-08-31

**Authors:** Alexandra L. Creavin, Cathy E. M. Williams, Kate Tilling, Karen Luyt, Nicholas Timpson, Julian P. T. Higgins

**Affiliations:** 1 MRC Integrative Epidemiology Unit, School of Social and Community Medicine, University of Bristol, Oakfield House, Oakfield Grove Clifton, Bristol, BS8 2BN UK; 2 Centre for Child and Adolescent Health, School of Social and Community Medicine, University of Bristol, Bristol, UK; 3 School of Clinical Sciences, University of Bristol, Bristol, UK

**Keywords:** Optic nerve, Retinal nerve fibre layer/retinal nerve fiber layer, Paediatrics/pediatrics, Ophthalmology, Premature/prematurity, Optical coherence tomography (OCT)

## Abstract

**Background:**

The parameters of the optic disc and peripapillary retinal nerve fibre layer (pRNFL) in premature children may vary with disease processes that contribute to visual impairment and blindness and so could be useful as an objective measure in at-risk children.

**Methods:**

A systematic review of current literature on the range of pRNFL and optic disc parameters in children aged less than 18 years, who were born before 37 weeks gestation, will be performed. The bibliographic databases MEDLINE, CINAHL, EMBASE, Scopus and Web of Science will be systematically searched. Where possible and appropriate, study-specific estimates will be combined using meta-analysis to obtain an overall summary estimate of pRNFL thickness and cup-disc ratio across studies, and results will be presented by age of population.

**Discussion:**

This review aims to improve understanding of what might be considered within/outside the range of normality for this high-risk group.

**Systematic review registration:**

The review is registered on PROSPERO: CRD42016037933

**Electronic supplementary material:**

The online version of this article (doi:10.1186/s13643-016-0319-0) contains supplementary material, which is available to authorized users.

## Background

### Rationale

Worldwide, around 10 % of babies are born prematurely (less than 37 weeks gestation), and premature birth is the leading cause of death in those under the age of 5 [1]. Children born prematurely are at a high risk of visual impairment [2], with around 4 % experiencing visual impairment and 1 % experiencing blindness at 2-year follow-up [3]. This blindness is frequently due to retinopathy of prematurity (ROP) and cerebral visual impairment (CVI). In some Western countries, optic nerve hypoplasia has overtaken ROP as the leading cause of infant blindness.

Since the advent of spectral domain portable optical coherence tomography (OCT), it has been possible to obtain detailed images of the retinal layers even in newborn children. Studies using this newly available technology have suggested that premature birth may be associated with optic nerve hypoplasia (odds ratio (OR) 3.47, 95 % confidence interval (CI) 2.25–5.35) [4] and have found that preterm children have a thinner peripapillary retinal nerve fibre layer (pRNFL) compared to term children. Park et al. and Wang et al. each found that compared to children born at term, preterm children had a thinner retinal nerve fibre layer (RNFL) in all sectors except the temporal RNFL, which may be thicker [5, 6]. One possible explanation for this may be the failure of migration of multiple retinal layers away from the fovea resulting in increased foveal and temporal RNFL thickness. Other explanations proposed are that the differing pRNFL anatomy relates to immature development of neurovascular structures [6, 7].

It is difficult to disentangle this finding from the relationship with retinopathy of prematurity [5, 8]. ROP stage has been shown to be inversely correlated with global average RNFL thickness, possibly related to laser photocoagulation [6, 9–11], and stage of ROP was inversely correlated with nasal RNFL thickness. Gestational age and birth weight were not found to be significant factors in this relationship [6].

Cerebral white matter damage (e.g. periventricular haemorrhage) is a common aetiology for CVI in premature children and can cause changes in the size of the optic cup and rim. In *adults*, white matter damage is also associated with changes in pRNFL thickness, which can be objectively measured easily using OCT [12–17]. Whether or not *children* with white matter injury have changes in pRNFL thickness is not well-established.

A number of small studies have investigated pRNFL thickness in children born prematurely, but to date, there is no review that combines these studies.

### Objectives

The objective of this review is to identify the parameters of the optic disc and peripapillary retinal nerve fibre layer (pRNFL) in children born prematurely, as measured by optical coherence tomography.

## Methods

### Eligibility criteria

#### Study characteristics

##### Population

The population is composed of children who are aged up to but not including 18 years at the time of assessment, who were born before 37 weeks gestation. Studies will be excluded if the results pertain only to a group of children with a specific pathology, e.g. children who have experienced facial trauma. Studies involving adult participants will be included if it is possible to extract data that is pertaining only to children.

##### Outcome

1. Peripapillary retinal nerve fibre layer (pRNFL) parameters (mean thickness, thickness of quadrants), measured using optical coherence tomography (OCT).
2. Optic disc parameters (disc area, vertical and horizontal disc height, cup size and resultant cup-disc ratio and neuroretinal rim area, obliquity) quantified by OCT.

Studies will be excluded if the measurements are not taken using OCT.

##### Types of study

Cross-sectional, cohort studies and control groups of case-control studies will be included. In the case of randomised controlled trials, it will be possible to include control-arm information and to include intervention group information, where the intervention would not affect the parameters of interest.

If sufficient population-based or prospective studies are available, these will be used in isolation. If it is necessary to include convenience or retrospective samples, these will be assessed for selection bias. Reviews, case reports and case series will not be included.

##### Report characteristics

Years considered

Databases will be searched from 1 January 1990 onwards, as OCT was not developed until 1990 [18].

Language

There will be no limitations on language as long as the title can be searched using English language keywords.

Publication status

Literature that is published online or in print will be included.

Other restrictions

Articles will only be included in the analysis where there are numerical measures of optic disc or pRNFL parameters.

### Information sources

#### Electronic databases

The following electronic databases were used: MEDLINE (via Ovid), CINAHL, EMBASE (via Ovid), Scopus and Web of Science.

#### Other

References lists will be searched (see search strategy below). The authors will also contact experts in the field for their unpublished data.

### Search strategy

Preterm, optic disc, retinal nerve fiber layer and paediatric keywords will be combined. Paediatric keywords were determined by compiling a combination of the Cochrane Child Health Field [19] with a University of Bristol paediatric search strategy which has been developed over a number of years. Terms specific to prematurity were not included in order to keep the search as broad as possible and avoid missing papers with premature infants as a subgroup.

### Example

(optic nerve? OR neuro?retinal rim OR nerve fiber layer? OR nerve fibre layer? OR RNFL? OR stratum opticum OR retinal nerve fiber? OR retinal nerve fibre? OR optic disc? OR optic disk? OR optic cup? OR cup-disc? OR cup-disk? OR nerve head? OR cupping) AND (spectral domain OR fourier domain OR optical coherence OR optical coherent?) AND (paediatric? OR pediatric? OR highschool? OR high school? OR secondary school? OR student? OR youth? OR young OR teen? OR prepubescent OR pre-pubescent OR pubescent OR puberty? OR preadolescent OR pre-adolescent OR adolesc? OR minors? OR juvenile? OR elementary school? OR primary school? OR schoolchild? OR schoolage? OR school-age? OR kids OR child? OR preschool? OR pre-school? OR nursery school? OR toddler? OR infant? OR babies OR newborn? OR neonat? OR girls OR boys) AND (gestation OR premature? OR preterm).

### Study records

#### Data management

Records will be managed using Covidence.

### Selection process

#### Screening

Studies identified by the search strategy will be screened in Covidence. Duplicates will be removed, and titles and abstracts will be screened by two members of the study team working independently. Disagreements will be resolved by discussion between them, with the option of further discussion with a third team member as required.

#### Eligibility

Full-text articles will be assessed for eligibility by two members of the study team working independently. Any disagreement between the two authors will be resolved by discussion between them, with the option of further discussion with a third team member as required.

Of the full-text papers selected for inclusion, the authors will search the reference lists of a random sample of five papers to screen for further work for inclusion.

### Data collection process

Primary and secondary outcome data will be extracted into an excel spreadsheet by two members of the study team working independently. Full data extraction will be duplicated for a random sample of 10 % of papers. Data extraction forms will be piloted on a sample of two papers. A proposed list of data to be extracted is given (Additional file 1).

Where required and feasible, the lead author will communicate with investigators of published studies in order to obtain or confirm data.

### Outcomes and prioritisation

Main outcomes

1. pRNFL: mean pRNFL thickness
2. Optic disc: cup-disc ratio

The outcomes will be summarised using means and standard deviations where possible and appropriate. If distributions of the outcome measurements are skewed, they may be reported using other statistics such as medians or geometric means, with interquartile ranges or ranges. Distributions will be summarised on the natural scale of the outcome, taking into account the possibility of skew.

### Additional outcomes

1. pRNFL: segmental pRNFL thickness (as quadrants or clock-hours depending on data available).
2. Optic disc: optic disc area, optic disc height, optic cup size; neuroretinal rim area; obliquity.
3. Global and central field macular thickness.

### Quality of individual studies

Data relating to the methodological quality of individual studies will be extracted as part of the data extraction process (see Additional file 1), including details of how individuals were selected into the study, the basis for exclusion from the study, scan quality and the use of published acquisition protocol such as the OSCAR-IB [20, 21]. The findings of this assessment will be used to inform a sensitivity analysis of high-quality studies.

### Data synthesis

Tables will be compiled giving descriptive information for each included study. These will describe the population examined, the examination protocol used, including machine make and model, and the baseline characteristics of participants.

A descriptive and graphical presentation of the individual study estimates of main outcomes will be given to include means and standard deviations with different makes and models of machines highlighted. Measurements made using time domain and spectral OCT devices and different makes or models of OCT machines will be compared using sensitivity analyses. If sufficient studies have used the same brand and model of an OCT machine in children of a comparable age, study-specific estimates will be meta-analysed to obtain an overall summary estimate of pRNFL across studies, by age of population. Subgroup analyses will be undertaken for children of different ethnicities, and adjustment will be made for ROP status. pRNFL quadrant data will be compared and likewise for clock-hour sectors, unless it is possible to reliably assign the clock-hours to a quadrant.

### Report of the review

The report of the review will follow the Preferred Reporting Items for Systematic Review and Meta-Analysis (PRISMA) guidelines. A PRISMA Protocol (PRISMA-P) checklist is included with this manuscript along with a list of data collection points (see Additional file 2).

## Discussion

This review aims to improve understanding of what might be considered within/outside the range of normality for this high-risk group. There is a reasonable risk that the number of papers covering this topic will be small and studies identified may vary greatly in methodology, particularly in terms of machines and software used. This heterogeneity will need to be taken into account in any conclusions drawn. However, even identification of an inadequate amount of data from which to draw conclusions would be important as it would highlight the need for work in this important area.

## Additional files

Additional file 1: Proposed list of data for extraction from full-text articles. (DOCX 15 kb) Additional file 2: PRISMA-P checklist. (DOCX 16 kb)

## Abbreviations

OCT: Optical coherence tomography
pRNFL: Peripapillary retinal nerve fibre layer
