# Supplementary material for: The range of peripapillary retinal nerve fibre layer and optic disc parameters, in children aged up to but not including 18 years of age who were born prematurely: protocol for a systematic review
Source: Syst Rev. 2016 Aug 31;5(1):144. doi: 10.1186/s13643-016-0319-0 (PMC5006449; doi:10.1186/s13643-016-0319-0)
Supplement: Additional file 1: — Proposed list of data for extraction from full-text articles. (DOCX 15 kb) [file 13643_2016_319_MOESM1_ESM.docx]

Data collection points

| **Data collection points** |
| --- |
| Population  Recruitment source: Population / clinic / school; Prospective/Retrospective  Number of children recruited (incl n female, n male)  Age (and years of birth) at different points of data collection  Gestation of children at birth  Birthweight of participants  Presence and grade of ROP  Country of recruitment and Ethnicity of participants  Are the children part of a named study?  Socioeconomic status |
| Years of data collection and number of waves of data collection |
| Number excluded  Were children excluded based on family history of eye disease?  Was the fundus examined? AND Were children with an abnormal fundus excluded?  Was the child refracted? AND Were children with a high refractive error excluded?  Was an orthoptic assessment performed?  Was visual acuity measured? AND Were children with a low visual acuity excluded? – at what level of VA?  Were children with a known diagnosis of eye disease or cerebral disease identified and/or excluded?  Other reasons for exclusion |
| Examination method  What OCT machine was used? (Make, model, time/spectral domain)  Who operated the OCT machine? AND was this to a predetermined protocol?  Were measurements automated, semi-automated or manual, and what software was used?  Was the OCT examination on a dilated pupil?  Was the image centred on the optic disc?  Were efforts made to exclude poor quality images? Specifically signal strength |
| Outcome measures (all mean, sd, median, n, range, units, by gender/age/eye/ethnicity)  Was analysis performed on one of the two eyes per person?  Average pRNFL  Peripapillary RNFL thickness by optic nerve sector: Temporal, Inferior, Nasal, Superior or by clock hour  Optic disc area; Horizontal and vertical disc diameter  Optic rim area  How was the cup defined?  Optic cup area; Cup depth; Horizontal and vertical cup diameter  Horizontal and vertical cup-disc ratio  Macular thickness: Global macular thickness, central field thickness, total macular volume |
| Is there a conflict of interest? |

Quality criteria

**A**

1. Population-based
2. Prospective
3. Known ophthalmic disease excluded / eye examination done and gross abnormality excluded
4. Signal strength 6 or more (where machine has this capacity)
5. Known cerebral/neurodevelopmental disorder excluded
6. OCT measurement involved use of a predetermined protocol
7. Study used a data coordinating center or published acquisition protocol
